# Anatomical variability and histological structure of the ulnar nerve in the Guyon’s canal

**DOI:** 10.1007/s00402-016-2616-4

**Published:** 2016-12-23

**Authors:** Paweł Depukat, Brandon Michael Henry, Patrick Popieluszko, Joyeeta Roy, Ewa Mizia, Tomasz Konopka, Krzysztof A. Tomaszewski, Jerzy A. Walocha

**Affiliations:** 1 Department of Forensic Medicine, Jagiellonian University Medical College, Krakow, Poland; 2 Department of Anatomy, Jagiellonian University Medical College, 12 Kopernika St, 31–034 Krakow, Poland

**Keywords:** Guyon’s canal, Ulnar canal, Ulnar nerve, Anatomy, Histology, Variations, Branching

## Abstract

**Objectives:**

The goal of our study was to analyze the prevalence of variations, branching patterns, and histology of the ulnar nerve (UN) in Guyon’s canal to address its importance in hand surgery, particularly decompression of the UN.

**Methods:**

Fifty fresh cadavers were dissected bilaterally, and the nerve in the area of Guyon’s canal was visualized. Samples for histology were also taken and prepared. The collected data were then analyzed.

**Results:**

Morphometric measurements of the hands and histological studies were not found to have significant differences when compared by left or right side or by sex. Three major branching patterns were found, with division into deep and superficial UN being the most common (85%). Additional findings included a majority (70%) presenting with a cutaneous branch within the canal and/or with an anastomosis of its distant branches with those of the median nerve (57%).

**Conclusion:**

The UN is most commonly found to divide into a superficial and deep ulnar branch within Guyon’s canal. However, additional branches and anastomoses are common and should be taken into careful consideration when approached during surgery in the area, particularly during decompression procedures of Guyon’s canal.

## Introduction

The Guyon’s Canal, also known as the ulnar canal, is a fibro-osseous tunnel located on the anteromedial side of the wrist, extending from the proximal end of the pisiform to the level of the hook of hamate [1]. This canal was first described in 1861 by Guyon [2] as an intra-aponeurotic compartment with its anterior wall being formed by a fibrous layer and its posterior wall being formed by the anterior carpal ligament.

Guyon described the canal as having a medial wall formed by the pisiform, coated with aponeurotic tissue proximally and fascia covering the hypothenar eminence distally [3]. Cobb et al. [4] described the lateral boundary of the Guyon’s canal as extending to, but not attaching to the hook of hamate, thus allowing for the ulnar artery and sensory components of the ulnar nerve (UN) to take a radial course in relation to the hook of hamate. The roof of the Guyon’s canal is formed by the distal extension of the antebrachial fascia, also known as the palmar carpal ligament, and adipose tissue. However, the hook of hamate does not form a true lateral wall for the canal [3, 4]. The lateral border of the canal is also formed partially by the insertion of the palmaris brevis muscle into the flexor retinaculum [4].

Although the terminology “Guyon’s canal” is widely accepted, there have been several proposals of alternate names of the canal in the literature. McFarlane et al. [5], who observed the palmaris brevis more distal but in the same position as Guyon, along with Enna et al. [6] suggested the term piso-hamate tunnel. Denman [7] noted that the ulnar carpal space passes beyond the level of the hook of hamate and that the palmaris brevis muscle forms the radial boundary of the space upon joining the flexor retinaculum. Therefore, he concluded that this region should be referred to as the piso-retinacular space and the passage of the deep branch of the UN be called the piso-hamate tunnel [4].

The Guyon’s canal and its anatomy are important in understanding the diagnosis and treatment of ulnar tunnel syndrome, also known as Guyon’s canal syndrome [3]. The compression of the UN within the Guyon’s canal produces a wide range of symptoms, including wrist pain radiating to the ulnar two digits associated with motor and sensory deficits [3]. Accessory muscles are the most common anatomical variations within the Guyon’s canal which might contribute to the symptoms of ulnar tunnel syndrome [3]. The symptoms might also be associated with the piso-hamate hiatus located between the piso-hamate ligaments and fibrous arch at the origin of the hypothenar eminence, a site where the deep branch of the UN might be compressed [3]. Furthermore, anatomical knowledge of the canal and the UN can be critical in surgical procedures of the hand [8–10].

Taking into account the clinical importance of UN compression in the Guyon’s canal and the anatomical variability of the canal, this paper aimed to: (1) identify where the UN splits into its superficial and deep branches and the distance from the pisiform to its point of branching; (2) identify the distribution and variations in the branches of the UN; and (3) evaluate where the palmar cutaneous branch leaves the Guyon’s canal; and (4) histologically evaluate cross sections of the UN.

## Materials and methods

### Cadaveric dissection

A total of 50 fresh cadavers (43 male, 7 female) between the ages of 29 and 100 years were dissected bilaterally at the Department of Forensic Medicine, Jagiellonian University Medical College, Krakow, Poland. No pathology or history of trauma was noted in the upper limbs of any of the cadavers. The area of the wrist was prepared and dissected to visualize the UN and the Guyon’s canal. A lateral incision was made parallel to the flexor carpi ulnaris muscle, starting at a point 1/3 of the way from the distal end of the forearm. The incision was continued in the shape of a “Z” from the wrist furrows and extended along the axis of the fourth metacarpal.

The flexor carpi ulnaris muscle, along with tendons, muscles, and subcutaneous fat, was removed to better visualize the UN, its branches, and the Guyon’s canal. The following morphometric parameters were measured: width of the wrist, distance between the distal ends of the second and fifth metacarpal, and the distance between the proximal end of the pisiform and the interdigital point between the fourth and fifth phalanx. After all measurements were obtained and a section of the UN was removed for histology, the incision was closed using a running intradermal suture.

### Histology

The UN in the region of the wrist was removed and fixed using a 10% solution of formaldehyde for 2–5 days. After fixing, a fragment of the trunk was taken 0.5 cm proximal to the splitting of the nerve. The sample was dehydrated in ascending concentrations of alcohol (50–96%), submerged and fixed in paraffin, sectioned (4 um), stained with hematoxylin and eosin (H&E), and assessed. A 100× magnification was used to count the nerve bundles, and the morphometric measurements were obtained using ImageJ (version 1.38).

### Statistical analysis

Statistical analysis was performed using Statistica 10.0 PL by StatSoft Poland. When appropriate, the mean, median, mode, and standard deviation were calculated. To determine if the data were normally distributed, the Shapiro–Wilk normality test was applied. Normally distributed data were analyzed using the student *T* test, while non-normally distributed data were analyzed using the Mann–Whitney *U* test. A *p* value of <0.05 was considered statistically significant.

### Ethical approval

The research protocol of this study has been approved by the Jagiellonian University Bioethics Committee (Registry No. KBET/118/B/2007). The study was performed in accordance with the ethical standards established in the 1964 Declaration of Helsinki and its later amendments.

## Results

### Morphometrics of the hand

Gross morphometric measurements of the hand according to gender are presented in Table 1. Our analysis showed that male cadavers in general had wider wrists, longer and wider metacarpals, and longer Guyon’s canal when compared to female cadavers. However, the distance from the branching point of the UN to the proximal end of the pisiform was longer in females, with a mean value of 2.6 ± 0.33 cm vs males (2.3 ± 0.80 cm, *p*-value 0.0861).

**Table 1:** Gross morphometrics of the hand (men vs women)

Measurement	Men				Women				*p* values
	*N* (number of hands)	Mean (cm)	Median (cm)	SD (cm)	*N* (number of hands)	Mean (cm)	Median (cm)	SD (cm)	
Width of wrist	43	11.50	11.60	0.89	7	9.60	9.00	0.92	<0.005
Width at metacarpals	43	16.20	16.20	0.93	7	13.60	13.60	0.79	<0.005
Length of metacarpal	43	16.30	16.20	1.33	7	15.20	15.40	0.63	0.053
Length of Guyon’s canal	43	4.50	4.60	0.51	7	4.00	4.00	0.21	<0.005
Distance from the branching point of the ulnar nerve to the proximal end of the pisiform	43	2.30	2.30	0.80	7	2.60	2.70	0.33	0.086

Morphometric measurements of the hand were also analyzed according to side and are presented in Tables 1 and 2. Our results showed that in general, all parameters measured were very similar on both sides with the *p* values of all measurements showing no statistically significant differences.

**Table 2:** Gross morphometrics of the hand (left vs right)

Measurement	Left				Right				*p* values
	*N* (number of hands)	Mean (cm)	Median (cm)	SD (cm)	*N* (number of hands)	Mean (cm)	Median (cm)	SD (cm)	
Width of wrist	50	5.60	5.70	0.57	50	5.64	5.70	0.57	0.75
Width at metacarpals	50	7.86	7.95	0.66	50	7.98	8.10	0.66	0.37
Length of metacarpal	50	8.07	8.00	0.67	50	8.06	8.00	0.66	0.94
Length of Guyon’s canal	50	2.23	2.30	0.26	50	2.23	2.20	0.26	0.95
Distance from the branching point of the ulnar nerve to the proximal end of the pisiform	50	1.19	1.30	0.50	50	1.03	1.15	0.50	0.05

### Branching patterns

**Fig. 1:**
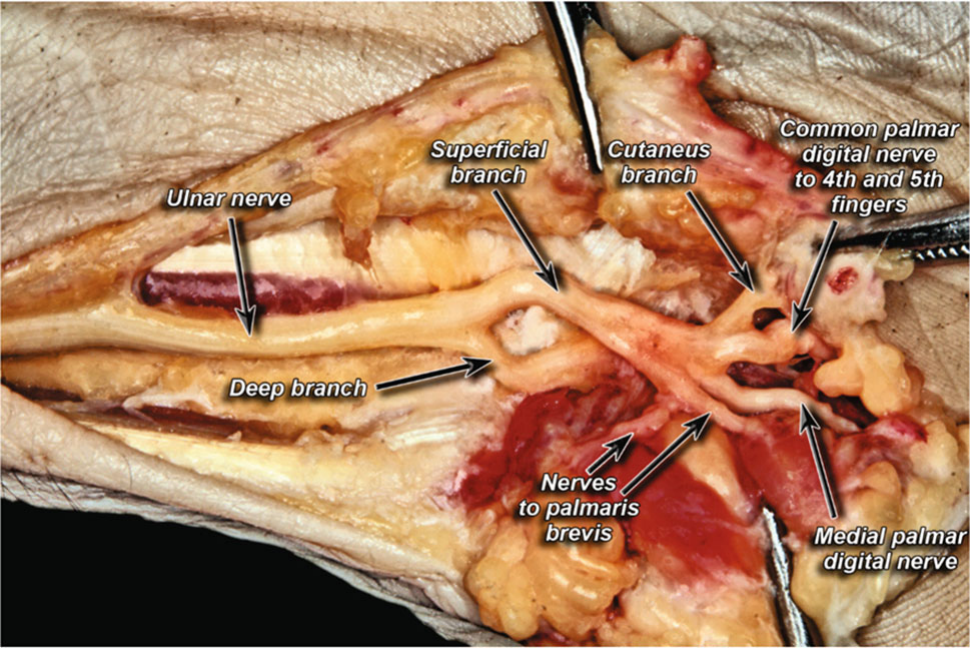
Classic division of ulnar nerve into deep and superficial branches

**Fig. 2:**
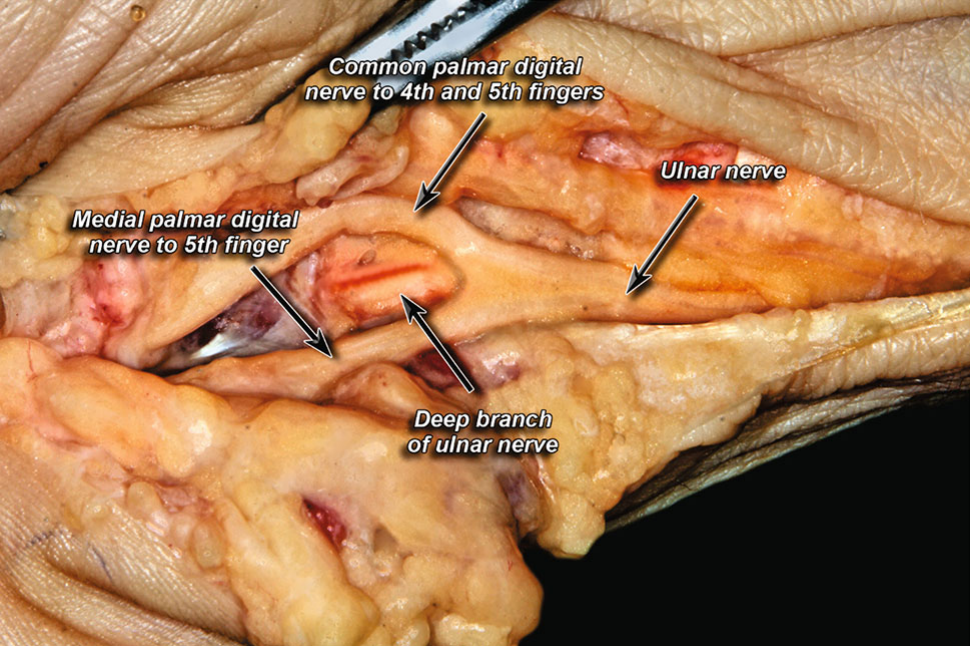
Trifurcation pattern of ulnar nerve

**Fig. 3:**
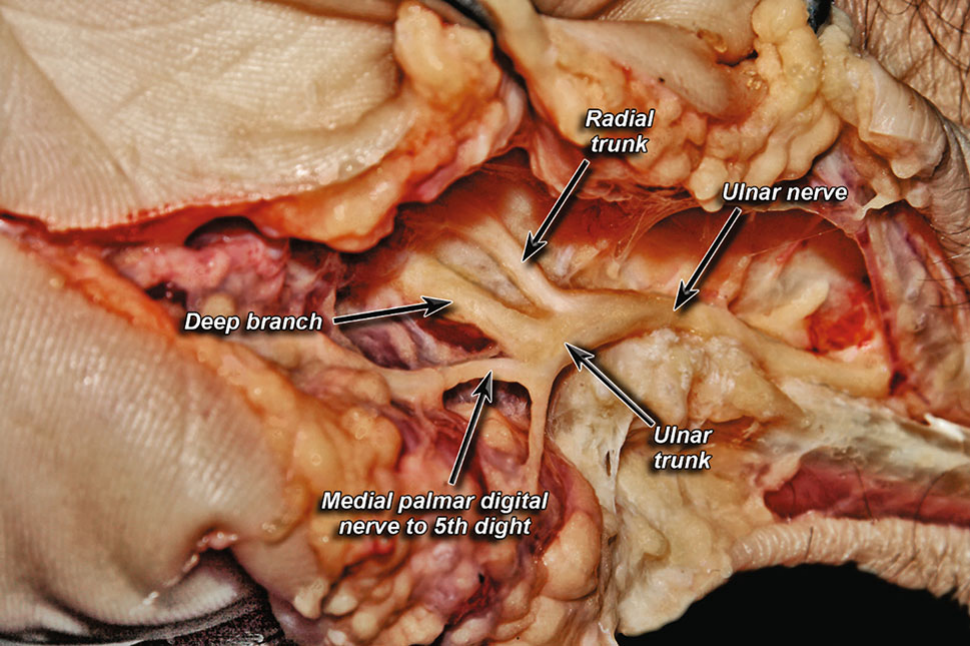
Division into a radial trunk and ulnar trunk

The dissection of the UN in the Guyon’s canal showed that the UN most commonly branched into two branches—a superficial branch and a deep branch in 85% of cases (Fig. 1). Trifurcation of the UN into a deep branch, a common palmar digital nerve (to digits 4 and 5), and a medial palmar digital nerve (digit 5) was seen in 13% of cases (Fig. 2). Finally, a division of the UN into a radial trunk and ulnar trunk was seen in only 2% of cases (Fig. 3). When compared by side, 78% of all the cadavers studied showed symmetry in their branching patterns. Asymmetry was only seen in one female, leading to 86% incidence of symmetry in females. Males on the other hand exhibited symmetry 77% of the time.

### Prevalence of a cutaneous branch within Guyon’s canal

**Fig. 4:**
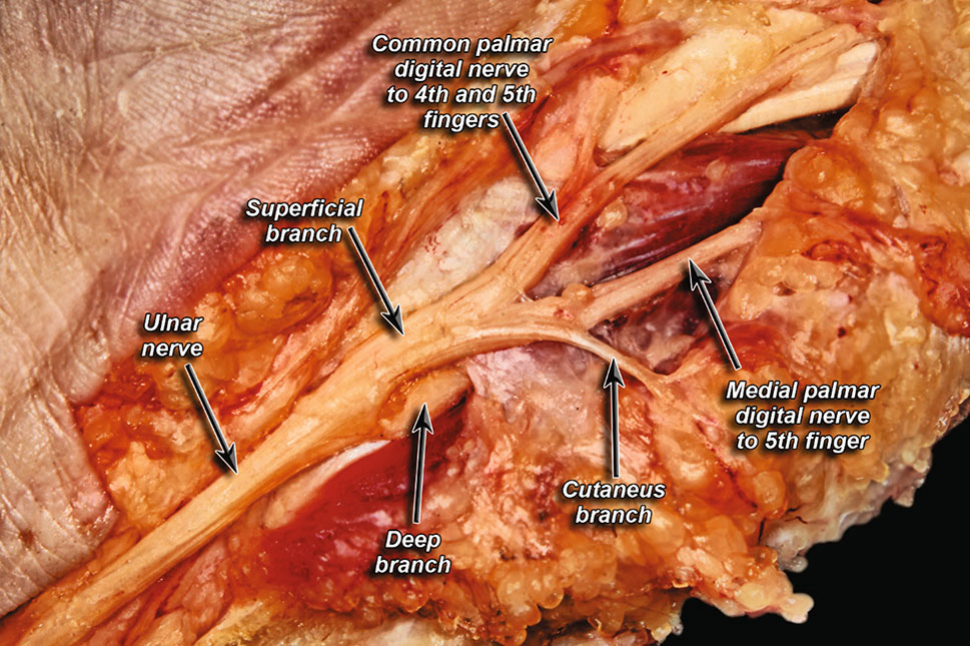
Presence of a cutaneous branch within Guyon’s canal

A cutaneous branch within the Guyon’s canal, branching from the superficial branch of the UN, was present in 70% of hands (Fig. 4).

### Anastomoses between branches of the ulnar nerve and between the ulnar and median nerves in the hand

**Fig. 5:**
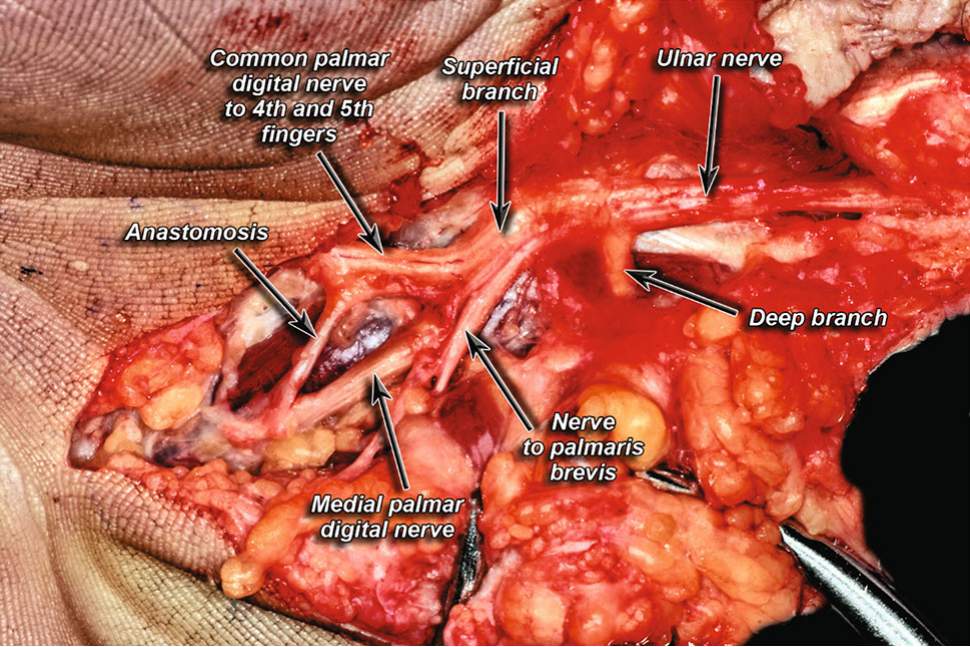
Anastomosis between digital branches of ulnar nerve

**Fig. 6:**
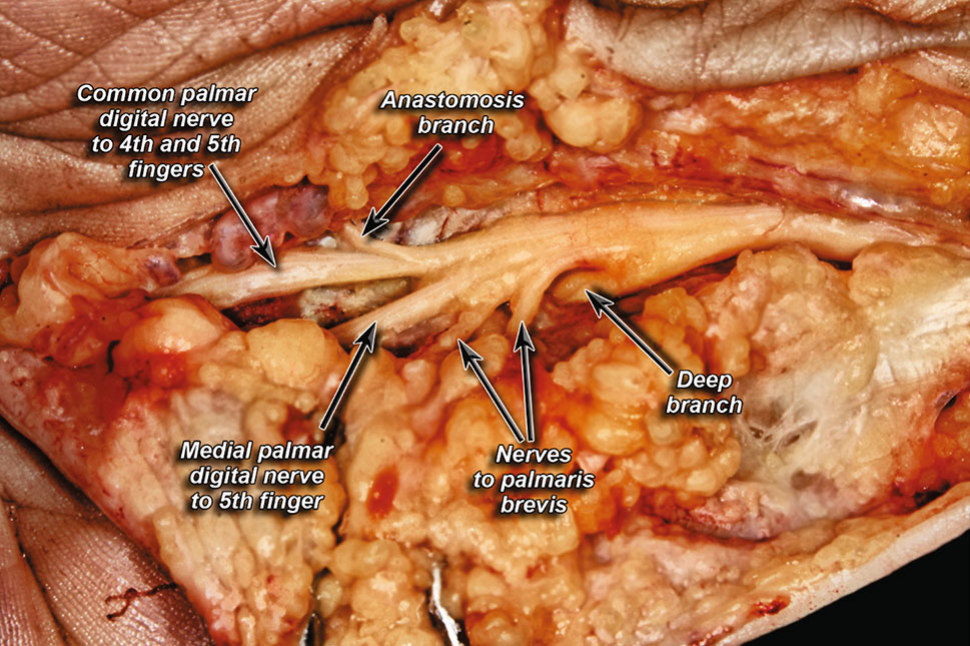
Anastomosis between branches of the ulnar nerve and median nerve

Anastomoses between the branches of the UN were present in 6% of cases (Fig. 5). Anastomoses between branches of the UN and MN, specifically between the common palmar digital nerves (to digits 4 and 5) and the lateral proper palmar digital nerve (to digit 4), were seen in 57% of cases (Fig. 6). Only 6% of the specimens had anastomoses between the UN itself.

### Muscular branches of the ulnar nerve to the palmaris brevis muscle

**Fig. 7:**
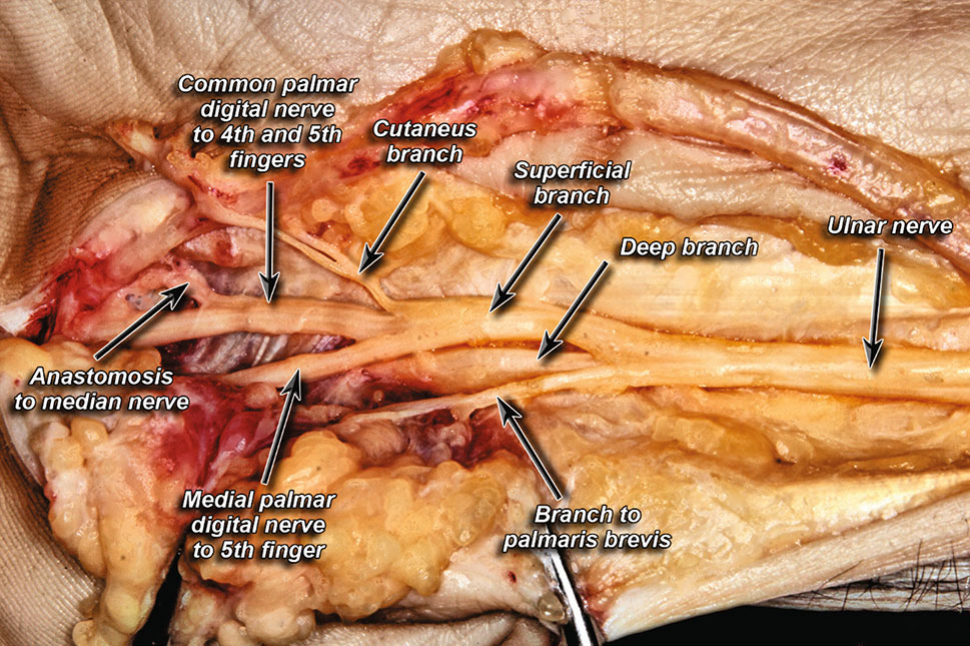
Branch to palmaris brevis muscle

The palmaris brevis muscle was most commonly supplied by one muscular branch from the superficial branch of the UN (52%) (Fig. 7) and was least commonly supplied by one muscular branch from the main branching point of the UN (6%). No muscular branches to the palmaris brevis muscle were seen in 34% of cases. Two branches from the superficial branch and one branch from the main branching point were seen in 8 and 6% of the specimens, respectively.

### Muscular branches of the ulnar nerve to the hypothenar eminence muscles

The hypothenar eminence muscles were most commonly supplied by one branch from the main branching point of the UN or one muscular branch from the ulnar trunk, both of which were seen in 9% of cases. The absence of muscular branches to the hypothenar eminence muscles was noted in 78% of hands. The least common patterns were two branches from the ulnar trunk and a configuration with one branch from the main branching point and one from the ulnar trunk, both occurring in 2% of the specimens.

### Histological structure of the ulnar nerve

Histological morphometric measurements according to gender are presented in Table 3. Similar to the gross morphometric measurements of the hand, our results did not reveal any statistically significant differences between the UN of male and female cadavers.

**Table 3:** Histologic morphometrics (men vs women)

Measurement	Men				Women				*p* values
	*N* (number of hands)	Mean	Median	SD	*N* (number of hands)	Mean	Median	SD	
Major axis (mm)	86	4.32	4.40	0.78	14	4.32	4.50	0.75	1.00
Minor axis (mm)	86	2.60	2.66	0.54	14	2.50	2.47	0.43	0.61
Cross-sectional area (mm^2^)	86	8.43	7.91	2.97	14	7.77	7.29	2.88	0.45*
Number of bundles	86	17.03	17.00	4.37	14	17.8	18.00	2.55	0.57

* Was evaluated with the Mann–Whitney test and is based on the median

Histological morphometric measurements according to side are presented in Table 4, and also showed no statistically significant differences between right and left hands.

**Table 4:** Histologic morphometrics (left vs right)

Measurement	Left				Right				*p* values
	*N* (number of hands)	Mean	Median	SD	*N* (number of hands)	Mean	Median	SD	
Major axis (mm)	50	4.45	4.52	0.76	50	4.20	4.14	0.77	0.09*
Minor axis (mm)	50	2.60	2.65	0.45	50	2.57	2.57	0.60	0.79
Cross-sectional area (mm^2^)	50	8.39	7.88	2.78	50	8.30	7.37	3.15	0.82*
Number of bundles	50	16.84	17.00	3.29	50	17.44	17.5	4.90	0.47

* Was evaluated with the Mann–Whitney test and is based on the median

**Fig. 8:**
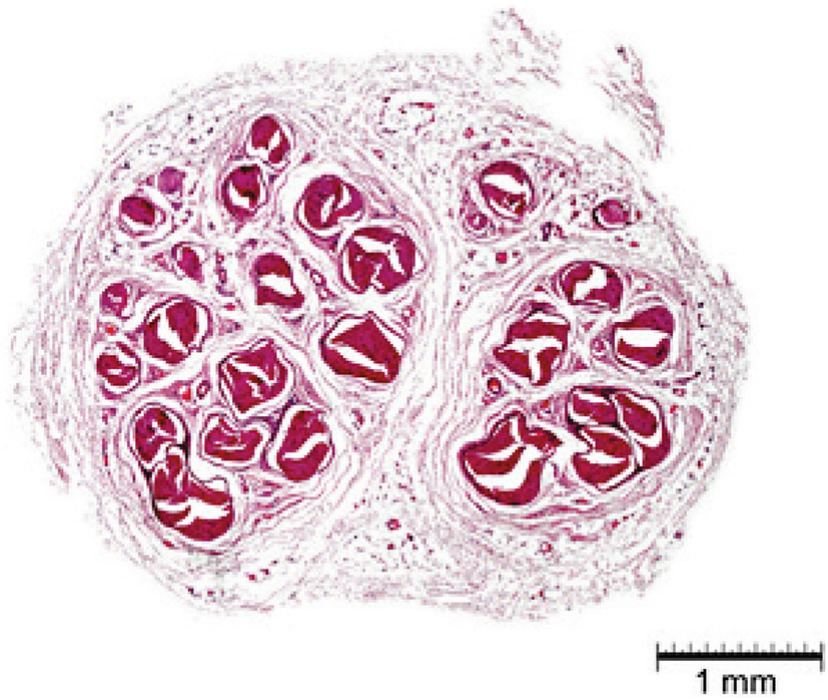
Histological preparation of ulnar nerve (H&E staining)

A sample of the histological preparations made is presented in Fig. 8.

## Discussion

The UN passes through Guyon’s canal as it makes its way from the forearm to the wrist, where it is prone to entrapment syndromes [11]. The goal of this study was to gather cadaveric data on the prevalence of variations of the UN and its branches within Guyon’s canal, its histological presentation, and any correlations of the nerve and the canal with the gross morphometrics of the hand.

We found that males generally had longer canals; however, in women, the UN travelled further before branching. No significant differences were noticed when comparing right to left sides. Likewise, in the histological study, no significant differences were noticed. The surgical significance of the findings above means that risk of injury in the area is not increased on either side, since findings are symmetrical. Since the success of nerve grafts greatly depends on the histological compatibility [12], our findings suggest that a nerve similar to the parameters described above can be used as a guide to picking a suitable graft. Women, however, would have a higher risk of a more complicated (sensory/motor) lesion if injury occurred, since there is a higher chance of encountering the combined, unbranched UN.

Branching patterns of the UN in this area commonly varied, with the most common bifurcation into superficial and deep branches constituting only 85% of the subjects. Trifurcation was the second most common with 13%. These branching patterns were found to be symmetrical in most cases (78%) suggesting that if surgery is needed on both hands in one patient, there is a strong chance that a similar pattern will be found. It was also noted that in 70% of specimens, the UN gave a cutaneous branch within Guyon’s canal, providing another nervous structure to be aware of during surgery so as to avoid accidental transection. Other common variations included anastomoses between the ulnar and median nerves at the level of the digital nerves to fingers four and five, which was observed in 57% of specimens. These findings are similar to findings in the previous literature on an anastomosis in the same area named the Berretini anastomosis which has a prevalence of 60.9% [13]. We would like to acknowledge that other variations in the anatomy of the UN are possible; however, they were not seen in the samples studied.

The contribution of the UN to the motor functions of the palmaris brevis and the muscles of the hypothenar eminence in the context of Guyon’s canal also varied greatly. Only 52% of the specimens had a branch to the palmaris brevis from the superficial branch of the UN, and 34% did not contribute to the motor innervation of the palmaris brevis. Though there were patterns of supplying the muscles of the hypothenar eminence, in 78% of cases, the UN did not contribute at all.

The limitations encountered in this study were mostly the small number of specimens studied. Furthermore, dissection did not spare the ulnar artery. Thus, estimation of anatomical relationship between the ulnar artery and the UN is not possible. Studies done in the past on Guyon’s canal have been performed using many different points of reference for measurement, making comparisons difficult. However, recent studies have shown similar findings in branching and anastomosing patterns. Murata et al. reported that in 86% of their samples, the UN bifurcated in Guyon’s canal, with trifurcation making up the remaining 14% [14]. They also reported that in 8.6% of the specimens, there was an anastomosis between the UN’s sensory branches to the fingers, compared to our 6% rate of occurrence [14].

The clinical importance of variations in the neurovasculature contained in Guyon’s canal is the role it plays in entrapment and UN neuropathies. Bozkurt et al. reported that the most common place for entrapment/compression within the canal is the distal end, and the most common cause is an anomalous slip of muscle [3]. He also suggests other causes, including lipomas, ganglia, and overuse of the hand; however, no such examples were seen in his study. Murata et al. also mentioned the importance of the existence of a fibrous fascial arch over the deep UN as a cause of entrapment. Though a majority of the specimens they studied included such an arch (30/35), in those that did not, they noted that further exploration and care should be taken to find the source of compression in this variant [14]. Most reports are individual case studies; however, in a retrospective study, Murata et al. found that a majority of cases of UN compression are idiopathic, with trauma being the second most common [15].

Further studies have described the space known as Guyon’s canal divided into three zones. Francisco and Agarwell [16] describe the three zones and their deficits as follows:

- Zone 1—the space past, the bifurcation of the UN into deep and superficial branches, presents with sensory, motor, or both types of deficits.
- Zone 2—the space surrounding the deep motor branch of the UN can present with paralysis of the intrinsic muscles and/or the hypothenar muscles.
- Zone 3—the space surrounding the superficial branch of the UN presents with only sensory deficits.

Such divisions have been described in the past as well; however, the terms proximal middle and distal were used to describe zones 1, 2, and 3, respectively [11]. The symptoms and zones, however, do correlate, and it was reported that just over half of the lesions observed were found in zone 2 [11].

Due to the variety of the UN within Guyon’s canal, the zones described above may not encompass all patients presenting with symptoms of UN entrapment. Certain anastomoses like those with the median nerve that was found in our study could present with more extensive deficits than those described above. For example, sensory deficits are common in the area between the middle and ring fingers in traumatic lacerations of communicating branches between the median and UN communicating branches [13]. Incidence of trifurcations would present with different zone patterns. Keeping the prevalence of these variations in mind, we would like to suggest as Ombaba et al. does that when a patient is considered for decompression surgery of Guyon’s canal that all zones be explored, regardless of patient presentation, to ensure success of the procedure [17]. Current literature for exploration of Guyon’s canal suggests exploring the canal from lateral to medial, or starting from zone 3 and proceeding up to zone 1 [1]. However, with such high prevalence of variation within the canal, we would like to stress that these suggestions should be followed to include all three zones, and not simply stop once the compression is thought to be found in a more distal zone. This way there can be little doubt as to whether the compression has been entirely eliminated.

## Conclusion

The UN in Guyon’s canal has been found to have a significant variance in its anatomy. The most common variation is the UN branching into a deep and superficial branch within Guyon’s canal. Common variants include trifurcation of the UN, anomalous small motor, and sensory branches, all of which give surgeons more structures to be aware of when operating in the area, particularly during UN decompression surgery. These variants can also alter the typical three-zone division of Guyon’s canal and the symptoms associated with each zone. A proper understanding of the possible variations can help surgeons to understand patients presenting with UN entrapment and ensure that proper planning and execution of decompression surgery resolves the patient’s symptoms.
